# Commonly consumed processed packaged foods in Bangladesh are unhealthy and their nutrient contents are not in conformity with the label declaration

**DOI:** 10.1002/fsn3.3772

**Published:** 2023-10-30

**Authors:** Nazma Shaheen, Abu Ahmed Shamim, Sohel Reza Choudhury, Sneha Sarwar, Md Musharraf Ashraf, Nisarga Bahar, Mohammad Abdullah Al Mamun, Sheikh Mohammad Mahbubus Sobhan, Md Joynul Abedin, Md Rizwanul Karim, Mohammad Robed Amin, Abdul Alim

**Affiliations:** ^1^ Institute of Nutrition and Food Science University of Dhaka Dhaka Bangladesh; ^2^ National Heart Foundation Hospital & Research Institute Dhaka Bangladesh; ^3^ Center for Non‐communicable Diseases and Nutrition, BRAC James P Grant of School of Public Health BRAC University Dhaka Bangladesh; ^4^ Non‐Communicable Disease Control (NCDC) Programme Directorate General of Health Services (DGHS) Dhaka Bangladesh

**Keywords:** Bangladesh, EU tolerance guideline, front of the pack labeling, health star rating, nutrition, packaged foods, processed foods, UK traffic light

## Abstract

The present study was undertaken to identify the major nutrient content in processed foods commonly consumed in Bangladesh, their label conformity healthiness, and percent daily nutrient contribution. Twenty‐four nationally representative composite samples were analyzed using AOAC and other standard methods. Results were compared with label information using a restrictive approach and EU tolerance guidelines. The healthiness of the products was evaluated in terms of the Health Star Rating (HSR) scheme and the UK traffic light labeling system. Among the analyzed samples, fried pulse, chanachur, lozenge, and fried peas had the highest amount of protein, fat, carbohydrates, and dietary fiber, respectively. Biscuits and milk chocolate had high levels of trans fatty acids (TFA) and saturated fatty acids (SFA). It was observed that around half of the products lacked information about saturated fatty acid (46%), followed by total dietary fiber and trans‐fat (38% each). Other information was missing in one‐fifth of the products, namely protein (17%), total fat (17%), available carbohydrate (17%), energy (17%), sugar (21%), and salt (21%). Label compliance analysis according to the restrictive approach revealed that none of the products accurately reported the salt, sugar, saturated fat, and trans fat content on the label. According to the EU tolerance guideline, approximately half of the products had protein (58%), fat (54%), and carbohydrate (42%) levels that fell within the EU tolerance limit. However, only around one‐third of the samples had sugar (21%), salt (38%), and saturated fat (33%) levels that met the EU tolerance limit. In terms of healthiness analysis, according to the HSR, the range of stars was between 0.5 and 2.5 of the foods where fried peas got the highest rating (2.5 stars), while in terms of the UK traffic light system, none of the samples got all green signals. The lozenge got green lights for fat, SFA, and salt contents. It was also found that consumption of 100 g of fried peas or pulse would exceed the acceptable daily limit of salt, sugar, and SFA compared to the daily maximum allowable intake for the 2000 kcal diet recommended by the WHO. However, according to the serving size, biscuits were major contributors of TFA, sugar, and SFA, whereas fried pulse was a key contributor of sodium/salt. Proper regulatory actions should be introduced to promote healthy processed foods with user‐friendly front‐of‐the‐pack labeling and monitor their quality to prevent non‐communicable diseases (NCDs).

## Graphical abstract

The present study was undertaken to test the hypothesis that the major nutrient contents in processed foods consumed in Bangladesh are in conformity with the label declaration. The healthiness of the products was evaluated in terms of the Health Star Rating (HSR) scheme and the UK traffic light labeling system. Proper regulatory actions should be introduced for monitoring and promoting healthful processed foods for the prevention of non‐communicable diseases (NCDs).

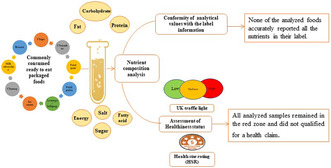

## 1 INTRODUCTION

With no time required to prepare, ready‐to‐eat processed foods suit the lifestyle of today's ever‐busy population. Moreover, global advancements in food preparation technology and open market policy have increased the availability and accessibility of packaged foods to the masses, gradually replacing home‐cooked meals and fresh fruit and vegetables in typical diets (Popkin et al., 2013). According to the existing literature, two‐thirds of dietary energy in high‐income countries is derived from processed foods (PAHO, 2015). This consumption pattern is also surging in low‐ and middle‐income countries (Baker & Friel, 2016).

Ultra‐processed foods, generally ready‐to‐eat and sold in convenient packages, are defined as “industrial products that are characterized by having high caloric density together with high levels of sugar, saturated fats, sodium, and insufficient levels of vitamins and minerals” (PAHO, 2015). Ready‐to‐eat processed or packaged foods comprise a wide variety of foods, including snacks, ready‐made meals, processed fruits and vegetables, dairy products, sugar‐sweetened beverages, and even formulae foods/baby foods (Hernandez Santana et al., 2020).

Bangladesh has seen an 8% average annual growth in the processed agro‐products and food processing sector, worth $2.2 billion (WEESMS, 2020). This growth demonstrates the increasing demand for processed foods in Bangladesh. Rapid urbanization, rising income, and availability are the major driving factors for the increased demand for processed ready‐to‐eat foods (Jones et al., 2017).

Studies have demonstrated that consuming processed and ultra‐processed foods leads to increased consumption of sodium, sugar, saturated fatty acids (SFA), and trans fatty acids (TFA), along with high energy but low dietary fiber consumption (Forouzanfar et al., 2015; Monteiro et al., 2010). Excessive consumption of processed foods is also associated with an enhanced risk of non‐communicable diseases, including diabetes, cardiovascular disease, and cancers (Forouzanfar et al., 2015). This circumstance demonstrates the urgency to control processed food intake.

Nutritional information in package labeling, particularly front‐of‐pack (FoP) labeling, is one of the most effective ways to control the consumption of ready‐to‐eat processed foods and their adverse public health effects (Pongutta et al., 2019). Labeling allows consumers to select the food based on their healthiness in terms of nutrient content (Azman & Sahak, 2014). However, nonconformities between the information presented on the label and the actual composition of the food are serious concerns in Bangladesh.

The present study was undertaken to test the hypothesis that the contents of carbohydrates, protein, fat, energy, total dietary fiber, sodium, total sugar, SFA, and TFA in commonly consumed processed foods in Bangladesh are in agreement with their label declaration. The study also reported the healthiness of processed foods in terms of the health star rating scheme and the UK traffic light system.

## 2 METHODS

### 2.1 Identification of commonly consumed processed foods

Data from previous cross‐sectional household and market surveys (Choudhury et al., 2021) conducted in 2019 was consulted to identify the most consumed processed foods and popular brands available in Bangladesh.

### 2.2 Sample collection

The enumerators visited one urban and two rural locations from each of the eight administrative divisions and collected samples of the predetermined list of commonly consumed processed foods from the grocery stores. This cross‐sectional study interviewed 948 individuals from 480 households in rural and urban areas in all eight administrative divisions of Bangladesh. Most frequently consumed commercially prepared ready‐to‐eat processed foods were identified based on their frequency of consumption (Choudhury et al., 2021). The foods included in the study were chips, chanachur, fried peas and pulses, noodles, biscuits, lozenges/lollipops, milk chocolate, chutney, and ice cream. Descriptions of the samples are provided in Table S1. Immediately after collection, samples were sent to the Institute of Nutrition and Food Science (INFS) laboratory for further sample preparation.

### 2.3 Sample preparation

Each sample was registered with a unique ID. Perishable foods were preserved in zip lock bags at −20°C until further processing. Other processed foods in intact packages were preserved in separate cardboard boxes at room temperature. A brand‐wise composite sample of each processed ready‐to‐eat food item was prepared by pooling at least 12 samples (ranging from 15 to 32 single samples to prepare a single composite) (Shaheen et al., 2013). Equal amounts of the same brand were mixed thoroughly, and the weights of the total mixture were recorded. Liquid nitrogen was added to immerse the mixture thoroughly, and the resulting crunchy mixture was then ground into powder using a grinder. The powder was finally transferred into aluminum foil and weighed.

100 g of each composite sample was wrapped in aluminum foil, placed in an airtight bag, and preserved at −20°C in a refrigerator until sent to a lab for analysis. For semi‐solid samples like ice cream, a composite sample was prepared by mixing equal amounts (by weight) of the same brand of samples collected from different retailers. Samples were sent to the laboratory, maintaining a cold chain.

### 2.4 Analysis of the composite samples

Samples were analyzed at FARE LABS Pvt. Ltd., India. The laboratory is accredited by the National Accreditation Board of Laboratories, India, and the Indian Body of International Laboratory Accreditation Cooperation. FARE LABS Pvt. Ltd. is also approved by the Food Safety and Standards Authority of India (FSSAI).

#### 2.4.1 Protein

Crude protein was estimated by the Kjeldahl method (No. 984.13; AOAC, 2000). The percentage crude protein in the sample was then calculated by multiplying the estimated nitrogen values with food‐specific Jones factors (Greenfield & Southgate, 2003).

#### 2.4.2 Fat

Fat was estimated by the continuous solvent extraction method (Soxhlet method) (no. 991.36 of AOAC, 2000) (Greenfield et al., 1992).

#### 2.4.3 Dietary fiber

The dietary fiber content was determined by the AOAC method (2000) using the total dietary fiber assay kit (enzymatic‐gravimetric method – Prosky (985.29)). Total dietary fiber content was then obtained by subtracting the protein and ash content from the weight of the residues (Cho et al., 1997; Greenfield et al., 1992; Horwitz & Latimer, 2000).

#### 2.4.4 Available carbohydrate

The total percentage of carbohydrate content in the food samples was determined by the differential method (Unzil et al., 2021). This method involved adding the total values of the sample's crude protein, lipid, dietary fiber, moisture, and ash constituents and subtracting it from 100. The value obtained is the percent carbohydrate constituent of the sample (Greenfield et al., 1992).

$$\%\text{carbohydrate}=100–\left(\%\text{moisture}+\%\text{dietary fiber}+\%\text{protein}+\%\text{lipid}+\%\mathrm{ash}\right).$$

#### 2.4.5 Energy value

The energy value of the samples was determined by multiplying the protein content by 4, carbohydrate content by 4, fat content by 9, and fiber content by 2. Then the total energy was obtained by summing all the values (Shaheen et al., 2013).

$$\text{Energy value}=\left(\text{Protein}\times 4\right)+\left(\text{Total carbohydrate}\times 4\right)+\left(\mathrm{Fat}\times 9\right)+\left(\text{Dietary fiber}\times 2\right).$$

#### 2.4.6 Fatty acid

Fatty acid profile was analyzed using Gas Chromatograph with FID, auto sampler (model Agilent 7890B) using column Supelco 2560 (100 m × 0.25 mm × 0.20 μm) Sigma. Infection temperature was 250°C at an injection gas (nitrogen) flow of 1 mL/minute. Blank and standard (FAME Mix GLC 674, manufacture: Nu‐Chek PREP; and Linoleic Acid Methyl Ester Isomer mix, manufacturer: Sigma Aldrich (Catalog no. CRM 4791) was run at the start of the chromatogram and after every 10 samples (AOAC, 2000).

#### 2.4.7 Sugar and salt

Total sugar was estimated by the Lane Eynon method. About 5 g of the sample was thoroughly mixed with 100 mL of hot water. The solution was filtered into a 250 mL volumetric flask. 100 mL of the water solution was added with 10 mL diluted HCl, heated for 5 min, and then cooled and neutralized with 10% NaOH. Phenolphthalein was added to the neutralized solution (Food Safety and Standards Authority of India, 2015) and titrated against Fehling's solution. Reading was calculated as follows:

$$\%\text{Total sugar}=\text{Factor}\;\left(4.95\right)\times \text{dilution}\;\left(250\right)\times 2.5\;\text{titer}\times \mathrm{wt}.\;\text{of sample}\times 10.$$

Salt was estimated by Mohr's titrimetric method. 5 g of sample was thoroughly mixed with 50 mL of distilled in a conical flask. 1 mL of potassium chromate was added to the mixture and titrated against standard silver nitrate solution with vigorous shaking. The appearance of reddish‐brown color indicated the endpoint (Food Safety and Standards Authority of India, 2015).

The percent NaCl was calculated from

$$\%\text{NaCl}=\frac{\text{Titer value}\times \text{Normality of the}\;\mathrm{AgN}O_{3}\times 58.4\times 100\;}{\text{Weight of the sample}\times 1000}$$

#### 2.4.8 Healthiness of processed packaged foods

##### Traffic light signal

Based on the salt, sugar, and saturated fats content, foods were classified using the “Guide to creating a front of pack (FoP) nutrition label for pre‐packed products sold through retail outlets” of the Department of Health in the UK (Department of Health, 2020). According to this guideline, red (high), amber (medium), and green (low) color coding is applied depending on the total fat, saturated fat, total sugar, and salt content of the food.

##### Australian heath star rating scheme

The Australian health star rating (HSR) scheme was developed by the Australian government and implemented voluntarily first in 2014. It is a front‐of‐pack interpretive nutrition labeling system that can guide consumers to make healthy choices. The system assigns ratings within 0.5–5 stars. The foods with 4 or fewer stars are ineligible to make a health claim. According to the algorithm, negative nutrients (overall energy, sodium, total sugar, and saturated fat) would lead to point subtraction, whereas positive nutrients (fruit and vegetable content, protein, fiber, etc.) would award additional points to the food. The measurement is done on their overall nutrient composition per 100 g or 100 mL for six major categories (Department of Health, 2020).

###### Analysis of label compliance

According to the Bangladesh Food Safety Act, 2017, it is mandatory to report energy, carbohydrate, protein, fat, saturated fat, sugar, and salt; this act also allowed voluntary reporting of fiber, and TFA on the label of the packaged food (BFSA, 2017). Bangladesh imposed restrictions on the level of TFA in foods in 2022 (Bangladesh Food Safety Authority (BFSA), 2021) So, as a scope of the study, the labels were analyzed for energy, carbohydrate, dietary fiber, fat, saturated fat, TFA, protein, salt and sugar.

###### Restrictive approach

In the case of analyzing percent variation, a restrictive approach was undertaken. The information on the label was collected and the percent variation was calculated as follows,

$\text{Percent}\;\left(\%\right)\text{variation}=\frac{\left(\text{analytical value}-\text{label value}\right)\times 100\;}{\text{label value}}$.

If the analytical result was an exact match of the label declaration, it was considered appropriate reporting. If the declaration was below or above the analytical value, it was considered underreporting or overreporting, respectively.

###### EU tolerance guideline

Label compliance was also measured according to the European Union tolerance guideline (European Commission, 2012) and an earlier literature (Albuquerque et al., 2020). To account for the measurement uncertainty, the EC guideline allows a lower and upper tolerable range of the declared value for comparing the analytic value. In the present study, the declared values were rounded and compared with the analytical values to determine if the rounded value fell within the tolerance limits, deviation beyond the tolerance limits indicating that the label value was out of tolerance (European Commission, 2012).

### 2.5 Percent contribution

According to the WHO, the regular allowable limits in a 2000 kcal healthy diet are SFA 200 kcal, TFA 2.2 g, sugar 25 g, salt 5 g, and sodium 2.3 g (WHO, 2020). Using the above WHO recommended values, the percent nutrient contribution from the studied foods was analyzed as follows,

$$\text{Percent}\;\left(\%\right)\text{nutrient contribution}=\frac{\text{Analytical value}\times 100\;}{\mathrm{WHO}\;\text{recommended value}}$$

### 2.6 Statistical analysis

All the data were analyzed in Microsoft Excel 2013 and SPSS version 20.0.

## 3 RESULTS

### 3.1 Obtained analytical values of macro and micronutrients

The nutrient contents of the most popular 24 brands from nine commonly consumed packaged food types (chips, chanachur, fried peas and pulse, noodles, biscuits, lozenge/lollipop, milk chocolate, chutney, ice cream) are shown in Table 1. It was found that lozenge brand 2 and fried pulse had the lowest (0.9 g/100 g) and the highest amount of protein (21.9 g/100 g), respectively. Fat and dietary fiber were absent in all lozenge brands, while chanachur brand 3 and fried peas had the highest level of fat (40.4 g/100 g) and dietary fiber (9.81 g/100 g), respectively. Chanachur brand 3 also had the highest energy value (563.71 Kcal/100 g). Available carbohydrate content was the lowest in ice cream brand 3 (11.39 g/100 g) and the highest in lozenge brand 1 (97.4 g/100 g). The maximum and minimum salt levels were found in fried pulses (7.2 g/100 g) and lollipops (0.1 g/100 g). The highest sugar content was found in lozenge brand 2 (49.8 g/100 g). The least sugar content, on the other hand, was found in vanilla ice creams brand 1 (2.50 g/100 g). Milk chocolates and biscuits had the highest level of SFA (26.07 g/100 g) and TFA (0.385 g/100 g), respectively. The lowest level of TFA was present in branded noodles (0.001 g/100 g). SFA was absent in lozenges and lollipops.

**TABLE 1:** Composition^a^ of commonly consumed processed ready‐to‐eat foods.

Types of foods		Protein (g/100 g)	Fat (g/100 g)	Carbohydrate (g/100 g)	Total dietary fiber (g/100 g)	Energy (kcal/100 g)	Sugar (g/100 g)	Salt (g/100 g)	SFA^b^ (g/100 g)	TFA^c^ (g/100 g)
Chips	Brand 1	5.2	26.8	56.9	3.2	495.9	7.2	2.4	12.5	0.1
	Brand 2	7.4	27.5	52.7	6.7	501.1	7.3	0.9	12.8	0.11
	Brand 3	4.8	18.9	63.9	4.4	454.1	7.04	2.9	9.4	0.12
Chanachur	Brand 1	14.0	36.1	41.5	3.8	555.0	7.4	0.4	14.9	0.11
	Brand 2	13.8	35.8	38.8	5.2	542.9	17.4	2.3	14.55	0.12
	Brand 3	13.8	40.3	33.89	4.9	563.7	10.5	2.4	18.07	0.12
Fried Peas	Brand 1	21.8	13.3	43.8	9.8	402.2	4.03	5.01	5.9	0.05
Fried pulse	Brand 1	21.9	16.1	45.9	3.4	423.6	2.4	7.2	7.9	0.09
Noodles	Brand 1	9.0	25.0	49.6	6.9	473.9	6.2	1.5	12.3	0.16
	Brand 2	11.6	0.2	65.6	9.1	328.4	5.9	2.8	0.04	0.001
Biscuits	Brand 1	8.0	18.1	60.8	4.4	447.0	27.04	3.2	9.3	0.06
	Brand 2	7.2	22.3	59.3	5.7	477.9	9.8	1.89	13.6	0.12
	Brand 3	6.5	21.2	65.1	2.4	482.2	28.0	0.51	12.5	0.39
	Brand 4	10.3	18.2	60.8	5.3	458.9	17.5	0.91	7.8	0.11
	Brand 5	11.3	17.6	60.4	4.03	453.8	16.2	2.1	11.1	0.04
	Brand 6	8.1	14.9	64.01	8.1	439.3	17.9	0.71	7.02	0.08
Lozenge	Brand 1	1.1	0	97.4	0	394.04	42.6	0.15	0	0
	Brand 2	0.9	0	96.9	0	391.3	49.8	0.81	0	0.062
Lollipop	Brand 1	1.3	3.9	93.3	0	413.6	35.5	0.1	3.5	0.22
Milk chocolate	Brand 1	8.9	27	51.4	8.1	500.3	7.4	2.2	26.1	0
Chutney	Brand 1	1.8	3.6	70.2	2.2	324.5	9.3	2.7	1.5	0.04
Ice cream	Brand 1	4.8	10.9	20.6	1.2	202.9	2.5	0.69	9.5	0.2
	Brand 2	4.2	15.6	18.2	0.9	231.8	2.8	0.7	11.02	0.2
	Brand 3	5.7	13.5	11.4	1.12	192.2	3	2.5	8.7	0.09

^a^ Composition was determined by chemical analysis of the composite sample.

^b^ Saturated fatty acids (SFA).

^c^ Trans fatty acids (TFA).

### 3.2 Presentation of nutritional information on the labels

According to the restrictive approach, when nutrient information on the label was compared with the analytical values, it was observed that around half (46%) of the products lacked information about saturated fatty acid, followed by total dietary fiber and TFA (38% each). The highest underreporting was observed in the case of salt (67%), TFA (58%), and total fat (54%). Around three‐fourth (71%) of the products over reported available carbohydrate contents in the foods (Table 2).

**TABLE 2:** Label compliance according to restrictive approach (*N* = 24).

Labeling status of nutrients	No reporting *N* (%)	Under reporting *N* (%)	Over reporting *N* (%)	Accurate reporting *N* (%)
Protein	4 (17)	13 (54)	7 (29)	0 (0)
Total fat	4 (17)	13 (54)	6 (25)	1 (04)
Available carbohydrate	4 (17)	3 (13)	17 (71)	0 (0)
Energy	4 (17)	8 (33)	12 (5)	0 (0)
TDF	9 (38)	10 (42)	3 (13)	2 (08)
Sugar	5 (21)	11 (46)	8 (33)	0 (0)
Salt	5 (21)	16 (67)	3 (13)	0 (0)
SFA	11 (46)	11 (46)	2 (08)	0 (0)
TFA	9 (38)	14 (58)	1 (04)	0 (0)

When the EU tolerance limits were taken into account for the labeled values, it was found that approximately half of the products had protein (58%), fat (54%), and carbohydrate (42%) levels that fell within the EU tolerance limit. However, only around one‐third of the samples had sugar (21%), salt (38%), and saturated fat (33%) levels that met the EU tolerance limit. (Table 3)

**TABLE 3:** Analytical value within the EU tolerance guideline.

Nutrient (*N* = 24)	EU range^a^		
	No reporting	Within tolerance	Outside tolerance
	*N* (%)	*N* (%)	*N* (%)
Protein	4 (17)	14 (58)	6 (25)
Fat	4 (17)	13 (54)	7 (29)
Carb	4 (17)	10 (42)	10 (42)
TDF	9 (38)	6 (25)	9 (38)
Salt	5 (21)	9 (38)	10 (42)
Sugar	5 (21)	5 (21)	14 (58)
SFA	5 (21)	8 (33)	11 (46)

^a^ To account for the measurement uncertainty, the European Commission guideline allows a lower and upper tolerable range for comparing the analytic vale with the label declaration (European Commission, 2012). In order to compare nutrient values declared on product labels with analytical values, the label information was rounded, (for example if the label value was between 11.45 g to 11.54 g, it would be considered as 11.5g). Then the upper and lower tolerable value was set at ±20%, resulting in an analytical value range of 9.15 g to 13.84 g per 100 g of food to be considered as within the tolerable limit. Next, the declared and analytical values were compared to determine if they fell within the tolerance limits; and any deviation in the declared values was noted, such as when the analytical value was outside the tolerance range of 9.15 g to 13.84 g per 100 g of food, indicating that the label value was out of tolerance.

### 3.3 Discrepancies between the obtained analytical values and label values for macronutrients

The restrictive approach also demonstrated marked discrepancies between the analytical values and label nutrient information for macronutrients. As shown in Table 4, the analytical values of protein, fat, carbohydrate, and energy content in chutney were, respectively, 805.0%, 1447.8%, 15.1%, and 31.4% higher than the respective label values. On the other hand, the analytical value of protein in chips (brand 1) was 54.8% lower than the label value. The analytical value of total dietary fiber in biscuits (brand 7) was 577.5% higher than the label value.

**TABLE 4:** Conformity of label information and analytical results for macronutrient content of processed ready‐to‐eat foods.

Food name	Sample	Protein		Fat		Available carbohydrate		Total dietary fiber		Energy	
		Label (g/100 g)	Difference from analysis result (%)	Label (g/100 g)	Difference from analysis result (%)	Label (g/100 g)	Difference from analysis result (%)	Label (g/100 g)	Difference from analysis result (%)	Label (kcal/100 g)	Difference from analysis result (%)
Chips	Brand 1	11.5	−54.8	17.2	55.9	66.45	−14.3	NA	*	471.0	5.3
	Brand 2	6.8	8.9	13.6	101.4	81.82	−35.7	0	*	504.6	−0.7
	Brand 3	10.0	−52.2	18.0	5.2	64	−0.1	NA	*	456.0	−0.4
Chanachur	Brand 1	17.0	−17.5	31.0	16.5	47	−11.6	1.0	284.0	535.0	3.7
	Brand 2	14.3	−3.8	35.7	0.4	46.43	−16.5	NA	*	607.1	−10.6
	Brand 3	17.6	−21.8	32.5	24.2	52.6	−35.6	4.0	23.0	502.0	12.3
Fried peas	Brand1	20.0	8.9	14.0	−4.6	54	−18.9	0	*	420.0	−4.2
Fried pulse	Brand1	23.0	−4.7	23.0	−29.7	50	−8.2	18.0	−80.9	500.0	−15.3
Noodles	Brand 1	10.8	−16.4	17.3	44.9	65.8	−24.7	7.2	−4.2	460.0	3.0
	Brand 2	9.7	19.3	0.9	−82.5	73.97	−11.4	11.7	−21.9	343.6	−4.4
Biscuits	Brand 1	7.0	15.3	17.0	6.5	75	−19.0	1.4	211.4	481.0	−7.1
	Brand 2	7.0	2.3	25.0	−10.7	64	−7.4	1.3	338.5	525.0	−9.0
	Brand 3	6.0	8.3	25.0	−15.2	60	8.6	NA	*	510.0	−5.5
	Brand 4	7.0	47.6	20.0	−8.9	72	−15.6	1.3	306.9	500.0	−8.2
	Brand 5	7.0	61.3	12.2	44.7	77.2	−21.7	0	*	450	0.8
	Brand 6	7.0	15.7	16.0	−6.6	76	−15.8	1.2	577.5	480	−8.5
Lozenge	Brand 1	0	*	0	*	96	1.5	0	*	384	2.6
	Brand 2	NA	*	NA	*	NA	*	NA	*	NA	*
Lollipop	Brand 1	0	*	0	*	96	−2.9	0	*	384	7.7
Milk chocolate	Brand 1	5.9	51.0	27.7	−2.5	64.1	−19.9	NA	*	530	−5.6
Chutney	Brand 1	0.2	805.0	0.23	1447.8	61	15.1	0		247	31.4

Abbreviations: *, Undefined; NA, Not available.

### 3.4 Discrepancies between the analytical values and label values for salt, sugar, SFA, and TFA

As shown in Table 5, the salt content of chutney brand 1 and the sugar content of chanachur brand 2 were, respectively, 8370.6% and 1644.0% higher than the respective label values. The highest discrepancy between analytical and label values in the SFA content was found in chips (brand 1). The obtained analytical value, in this case, was 780.2% higher than the labeled value (Table 5). The analytical value in the dairy milk chocolate was lower than the label value.

**TABLE 5:** Conformity of label information with obtained analytical results for salt, sugar, SFA, and TFA content of processed ready‐to‐eat foods.

Food name	Sample	Salt		Sugar		SFA		TFA	
		Label (g/100 g)	Difference from analysis result (%)	Label (g/100 g)	Difference from analysis result (%)	Label (g/100 g)	Difference from analysis result (%)	Label (g/100 g)	Difference from analysis result (%)
Chips	Brand 1	1.6	50.6	2.45	191.8	1.42	780.2	NA	*
	Brand 2	3.8	−97.6	0	*	0	*	0	*
	Brand 3	1.6	86.3	2.4	193.3	8	17.2	0	*
Chanachur	Brand 1	2	−80.0	0	*	3	395.3	0	*
	Brand 2	2	15.5	1	1644.0	2.86	408.7	NA	*
	Brand 3	NA	NA	NA	*	NA	*	NA	*
Fried peas	Brand 1	0.4	1045.1	0	0.00	4	47.1	0	*
pFried Pulse	Brand 1	1.6	346.9	2	22.0	4	97.3	0	*
Noodles	Brand 1	1.9	−20.3	1.6	286.9	8.17	51.0	0	*
	Brand 2	2.3	21.4	0	0.00	0	*	0	*
Biscuits	Brand 1	1.8	82.9	12	125.3	10	−7.1	0	*
	Brand 2	1.1	67.3	11	−10.6	13	4.8	NA	*
	Brand 3	0.2	240.0	18	55.7	12	4.1	0	*
	Brand 4	0.8	21.3	18	−2.9	7	11.4	0	*
	Brand 5	0.1	4120.0	17	−4.7	0	*	0	*
	Brand 6	0.5	42.0	18	−0.8	8	−12.2	NA	*
Lozenge	Brand 1	0.3	780.0	61.2	−87.9	16.9	54.3	0.1	−100
	Brand 2	0.01	757.1	56	−23.8	0	*	0	*
Lollipop	Brand 1		NA	NA	*	NA	NA	0	*
Milk chocolate	Brand 1	0.01	700.0	56	−36.6	0	*	0	*
Chutney	Brand 1	0.03	8370.6	53.6	−82.6	0	*	0	*

Abbreviations: *, Undefined; NA, Not available; SFA, Saturated fatty acids; TFA, Unsaturated fatty acids.

### 3.5 Healthiness of processed foods based on the health star scheme and the traffic light system

The health star rating scheme and the UK traffic light system were used to assess the healthiness of the processed foods included in the study. According to the health star scheme, the range of stars was between 0.5 and 2.5 of the foods, the highest rating was obtained by branded fried peas (2.5 stars). In terms of the UK traffic light system, none of the samples got all green signals. Only the lozenge sample got green lights in terms of fat, saturated fat, and salt contents (Table 6).

**TABLE 6:** Healthiness of the processed ready‐to‐eat foods available in Bangladesh based on fat, SFA, sugar, and salt content.

Name of the foods	Health star rating (number of stars)^a^	UK traffic light system^b^			
		Sugar (g/100 g)	Salt (g/100 g)	Saturated fat (g/100 g)	Fat (g/100 g)
Chips	0.5–2.0	7.2^a^	2.1^r^	11.6^r^	24.4^r^
Chanachur	0.5–1.5	11.8^a^	1.7^r^	15.8^r^	37.4^r^
Fried pea	2.5	4.0^g^	5.01^r^	5.9^r^	13.4^a^
Fried pulse	2	2.4^g^ ^r^	7.15^r^	7.9^r^	16.2^a^
Noodles	1–1.5	6.1^a^	2.2^r^	6.2^r^	12.6^a^
Biscuit	0.5–2.0	19.4^a^	1.6^r^	10.2^r^	18.7^r^
Lozenge	1–2	46.19^r^	0.48^g^	0^g^	0^g^
Lollipop	1.5	35.5^r^	0.14^g^	3.54^a^	3.95^a^
Milk Chocolate	0.5	7.4^a^	2.2^r^	26.0^r^	27^r^
Chutney	1	9.3^a^	2.7^r^	1.5^a^	3.5^a^
Ice cream	0.5–1.5	2.8^g^	1.3^a^	9.7^r^	13.3^a^

^a^ The foods having 4 stars or above are defined as healthy.

^b^ Total fat: low (<3 g/100 g), moderate (>3–17.5 g/100 g), high(>17.5 g/100 g); saturated fat: low (<1.5 g/100 g), moderate (>1.5 to ≤5 g/100 g), high (>5.0 g/100 g); salt: low (<0.3 g/100 g), moderate (>0.3‐ ≤ 1.5 g/100 g), high (>1.5 g/100 g); sugar: low (<5 g/100 g), moderate (>5.0 to ≤22.5.0 g/100 g), high (>22.5 g/100 g), ^a^ = Amber color; denoting a medium level of the nutrient, ^g^ = green denoting a low level of the nutrient, ^r^ = Red color; denoting a high level of the nutrient.

### 3.6 Contribution of 100 g of processed foods to dietary intake of sugar, salt, SFA and TFA

**FIGURE 1:**
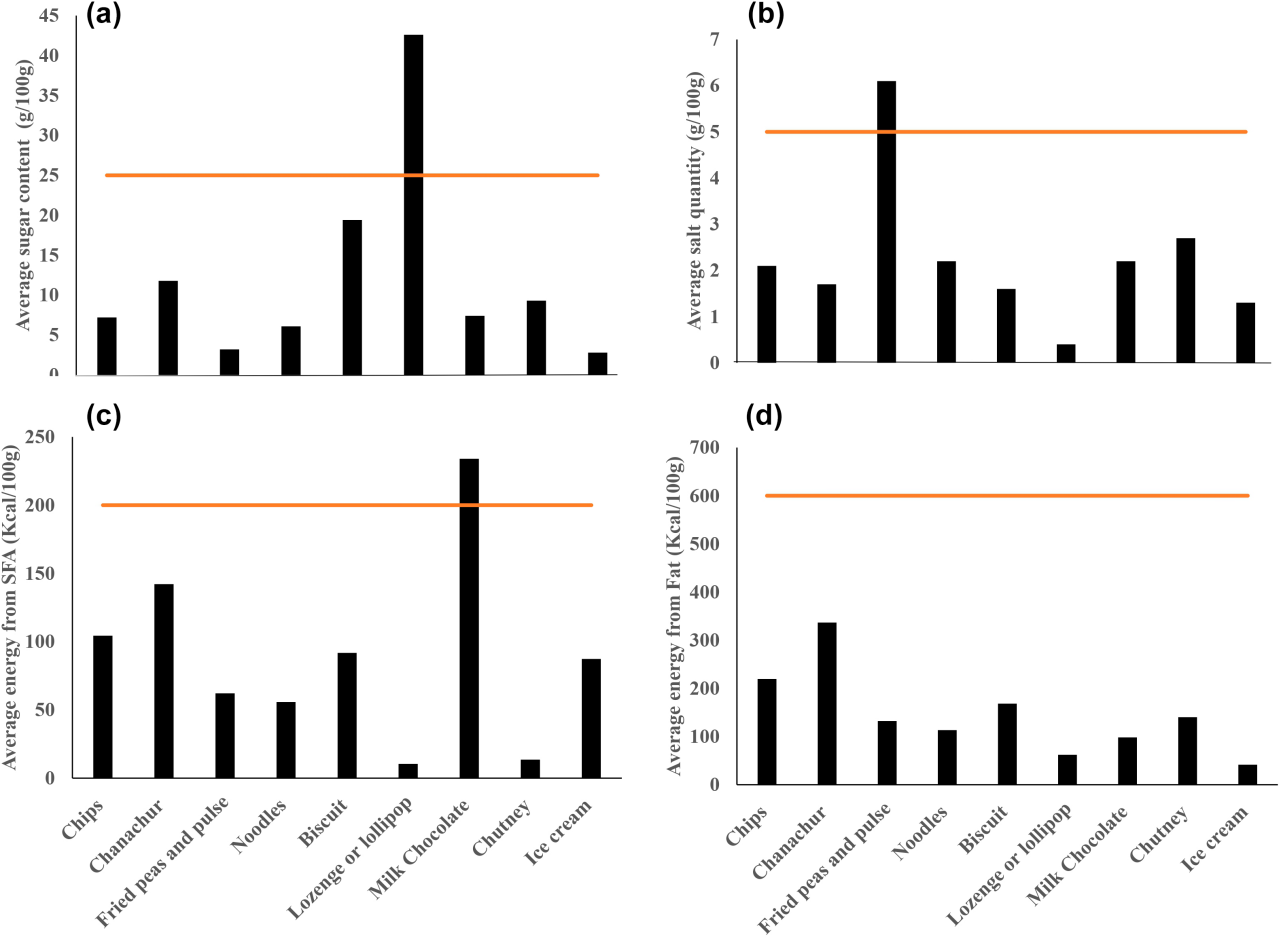
Contribution of 100 g of different groups of food to the daily intake of (a) salt, (b) Total kcal from total fat, (c) Sugar, and (d) Total kcal from saturated fatty acid (SFA). The horizontal line indicates the daily maximum allowable intake for the 2000 kcal diet recommended by the WHO.

The contribution of 100 g of processed foods to the intake of sugar, salt, saturated fat, and total fat in relation to the daily maximum allowable intake for the 2000 kcal diet recommended by the WHO is summarized in Figure 1. When analyzed against the WHO‐recommended daily limit of salt, sugar, and saturated fat intake, and it was found that 100 g of milk chocolate would exceed the saturated fat (234.9 kcal) intake limit. 100 g of lozenge would provide 43 g of sugar, far exceeding 25 g allowed in a healthy diet, whereas biscuits, chanachur, chutney, and milk chocolate could contribute a substantial share of the maximum allowable amount. The intake of 100 g of fried peas and peas would contribute 6.1 g of salt, more than the maximum allowable amount of 5 g. Chutney, milk chocolate, noodles, and chips could also contribute a substantial proportion of the maximum allowable amount of salt.

### 3.7 Contribution of per serving of processed foods to the daily intake of critical nutrients

The contribution of per serving processed foods to the dietary intake of sugar, salt, sodium, SFA, and TFA was analyzed. It was found that per serving of Biscuit accounted for the highest percentage of sugar (27.04%; brand 1), TFA (3.19%, brand 3), and SFA (38.85%; brand 5). Fried pulse was identified as the key contributor of salt (36%) and sodium (31.3%) (Table S2).

## 4 DISCUSSION

The present study was undertaken to determine the major nutrient content in processed foods commonly consumed in Bangladesh. The study also reported the percent daily nutrient contribution, healthiness, and conformity to label guidelines of these foods.

Among the analyzed samples, fried pulse, chanachur, lozenge, and fried peas were identified as significant sources of protein, fat, carbohydrates, and dietary fiber, respectively. Biscuits and milk chocolate had high levels of trans fatty acids (TFA) and saturated fatty acids (SFA). However, none of the analyzed food had more than 2% TFA. These findings are in agreement with earlier studies in Brazil and Spain which reported 0.86% TFA in biscuits and 0.68% TFA in bakery products, respectively (Dias et al., 2015; Ricardo et al., 2019). A recent study findings in India reported 1 bakery product and 3 deep fried products out of 75 samples to have the TFA content exceeding 2% (Joshee et al., 2019).

Under the scope of the study, labels were analyzed in terms of accuracy and completeness of the information. It was observed that around half of the products lacked information about saturated fatty acid, followed by total dietary fiber and TFA. Information about protein, total fat, available carbohydrate, energy, sugar, and salt was missing in one‐fifth of the products. Even though after having mandatory food labeling for specific nutrients, several nutrients information were missing.

Only presence of label information is not enough, it needs to be accurate. When the restrictive approach was applied for label analysis, it was found that only 4% and 8% of the products complied with their declared fat and fiber content, respectively. When the EU tolerance guidelines were applied, approximately half of the products met the labeling requirements for macronutrients. However, only one‐third of the products' declarations were within the tolerance limits for saturated fatty acids, salt, sodium, and sugar. These findings indicate poor implementation of the packaged foods labeling regulation.

In order to analyze healthiness, the UK traffic light system and health star rating scheme were applied. None of the foods in the study qualified for the healthiness claim as per the UK traffic light system, though some of the products received the green light for salt, sugar, TFA, and/or SFA contents. According to HSR, foods that receive 4‐star or higher ratings are considered eligible for health claims (Crino et al., 2018). None of the processed foods in the study met the above healthiness criteria and can be considered unhealthy. This finding was in conformity with a study of packaged foods and beverages from India; 54% of the analyzed food products scored within 0.5–1.5 stars, indicating they were not eligible for health claims (Jones et al., 2017).

Consumption of a serving of these foods can provide substantial portion of the WHO‐recommended maximum allowable limit of sugar, salt, and SFA for a healthy diet. Among the analyzed foods, one serving of biscuit was found to be a key contributor of sugar, SFA, and TFA, while fried pulse was a prime contributor of sodium/salt. However, one serving of the samples did not exceed the daily limit of these nutrients.

These findings have important implications for public health in Bangladesh. Consumption of high levels of salt, sugar, saturated fat, or TFA regularly can cause severe health hazards. For instance, high salt intake is directly related to elevated blood pressure (Cappuccio et al., 2019). Increased intake of SFA is significantly linked with elevated total cholesterol and an increased risk of cardiovascular diseases (Te Morenga & Montez, 2017). The World Health Organization recommended that energy intake from saturated fat should not exceed 10% of total energy intake (World Health Organization (WHO), 2020). Consumption of TFA above the critical safe limit may increase the risk of cardiovascular diseases such as atherosclerosis. However, the TFA content of all the products included in the present study was within this limit of 2% as recommended by the WHO (World Health Organization (WHO), 2020); and Bangladesh Food Safety Authority (Bangladesh Food Safety Authority (BFSA), 2021).

The limitations of the study include variations in sugar and salt estimation methods. This should be considered when interpreting the results. The copper reduction method was used to analyze sugar content, which might introduce a 2–4% variation in the estimated values (Rajakylä & Paloposki, 1983). Salt was assayed in this study using widely used Mohr's titrimetric method, which indirectly estimate it from chloride ion titrated with silver nitrate solution, and may underestimate total amount of salt in food, as salt may come from multiple sources namely preservatives, leavening agent, etc. (Chen et al., 2005). In spite of these limitations, the study is the first attempt to assess packaged processed foods' conformity to label guidelines in Bangladesh.

Bangladesh is undergoing rapid urbanization and economic development and, like many developing countries, is experiencing a shift of food preference from cereal‐based to processed foods (Waid et al., 2019). Consumption of processed foods starts from very early age in Bangladesh, a habit that might be continued during the adulthood; children (Jannat et al., 2020) and adolescents (Shamim et al., 2023) frequently consume sugar‐sweetened beverages and savory and sweet foods; so, it is important to protect the health of the vulnerable population through appropriated policy measures. Restrictions on advertising and imposing taxes were effective in reducing the consumption of unhealthy processed foods in many countries (Monteiro et al., 2010). Modifying ingredients for food production by lowering the quantity of salt, sugar, and saturated and TFA may also improve food quality and make the product nutritious and safe. Mandatory food labeling and the national ban on unhealthy packaged food can also be adopted to regulate the nutrients in foods (Ricardo et al., 2019). Consumer‐friendly front‐of‐the‐packet labeling can help the consumer avoid unhealthy packaged foods (Cecchini & Warin, 2016). Awareness programs for consumers and food producers regarding the health consequences of high sugar, salt, TFA and saturated fatty acid will also help reduce the consumption of these unhealthy foods.

## 5 CONCLUSION

Using nationally representative samples, our study showed that the overall healthiness of packaged foods and beverages in Bangladesh was poor and the nutrition information displayed was not enough for the consumers to make informed decisions. The compliance with the declarations was also poor. Each serving of these unhealthy packaged foods found to contribute considerable amount of RDA, which is also alarming. This characterizes public health issue as the Bangladeshi consumers are buying ever increasing amount of processed foods without access to basic and user‐friendly information about healthiness of the foods. It is expected that the policy makers, program managers, and the food regulating authorities will take initiatives to improve the quality of packaged processed foods and beverages to abate the burden of diet‐related non‐communicable diseases. Absence of mandatory declaration of TFA and fibers from the Packaged Food Labelling Act 2017 warrant immediate review. It is also essential to introduce a user‐friendly front‐of‐the‐pack labeling system to support consumers in decision making, especially to avoid excessive consumption of energy‐dense foods high in sugar, salt, saturated fat, and trans fat and poor in health‐promoting nutrients such as fiber.

## AUTHOR CONTRIBUTIONS

**Nazma Shaheen:** Conceptualization (lead); data curation (equal); formal analysis (equal); supervision (equal); writing – review and editing (equal). **Abu Ahmed Shamim:** Conceptualization (lead); data curation (equal); formal analysis (equal); supervision (equal); writing – review and editing (equal). **Sohel Reza Choudhury:** Conceptualization (lead); data curation (equal); formal analysis (equal); funding acquisition (lead); methodology (lead); project administration (lead); supervision (equal); writing – original draft (equal); writing – review and editing (lead). **Sneha Sarwar:** Conceptualization (equal); writing – original draft (equal). **Md Musharraf Ashraf:** Conceptualization (equal); writing – original draft (equal). **Nisarga Bahar:** Data curation (equal); supervision (equal); writing – review and editing (equal). **Mohammad Abdullah Al Mamun:** Data curation (equal); formal analysis (equal); project administration (lead); supervision (equal); writing – review and editing (supporting). **Sheikh Mohammad Mahbubus Sobhan:** Data curation (equal); supervision (equal); writing – review and editing (supporting). **Md Joynul Abedin:** Data curation (equal); formal analysis (equal); supervision (equal); writing – review and editing (supporting). **Md Rizwanul Karim:** Resources (equal); supervision (equal). **Mohammad Robed Amin:** Data curation (equal); formal analysis (equal); supervision (equal); writing – review and editing (equal). **Abdul Alim:** Data curation (equal); supervision (equal); writing – review and editing (equal).

## FUNDING INFORMATION

The present study was funded by the Directorate General of Health Services, Ministry of Health and Family Welfare, Bangladesh, under its Non‐Communicable Disease Control (NCDC) Program (Memo No. DGHS/LD/NCDC/Proc. Plan/GOB (Service)/2018‐2019/SP‐13/Negotiation/348, Date: 20.01.2019).

## CONFLICT OF INTEREST STATEMENT

The authors declare that they have no conflicts of interest.

## ETHICS STATEMENT

Ethical approval was obtained from the Institutional Review Board of the Institute of Health Economics (IHE‐IRB), University of Dhaka, which is approved by the U.S. Department of Health and Human Services Federal Wide Assurance (Ref. No. IHE/IRB/DU/44/2021/Final, Date: 30 December 2021)

## Supporting information


Tables S1–S2

## Data Availability

The data that support the findings of this study are available on request from the corresponding author. The data are not publicly available due to restrictions by the governmental funding agent, Non‐Communicable Disease Control (NCDC) Program, Directorate General of Health Services, Ministry of Health and Family Welfare, Bangladesh.
