# Supplementary material for: Commonly consumed processed packaged foods in Bangladesh are unhealthy and their nutrient contents are not in conformity with the label declaration
Source: Food Sci Nutr. 2023 Oct 30;12(1):481–93. doi: 10.1002/fsn3.3772 (PMC10804074; doi:10.1002/fsn3.3772)
Supplement: Supplementary file 1 — Tables S1–S2 [file FSN3-12-481-s001.docx]

### Supplementary table 1: Description of most commonly consumed processed foods

| **Name of samples** | **Description** | **Brand collected** | **Number of samples used to make composite** |
| --- | --- | --- | --- |
| Chips | Chips of various brands sold in plastic package. Produced by large producers and local (informal) producers | Brand-1 | 32 |
|  |  | Brand-2 | 23 |
|  |  | Brand-3 | 19 |
| Chanachur | Chanachur is a popular snack made from a mixture of different components, including fried noodles made from pulse flour, wheat flour chips, fried and puffed pulses, rice, and peanuts. The ingredients used can vary depending on the market and price, with small producers in rural areas typically using fewer components than branded chanachurs. Spices and condiments are added for flavoring.. | Brand-1 | 26 |
|  |  | Brand-2 | 27 |
|  |  | Brand-3 | 15 |
| Noodles | Noodles refer to a food item that is made from dough without yeast, which is flattened, cut, stretched or extruded into long strips or strings. They are typically prepared by boiling in water, sometimes with added salt or cooking oil. | Brand 1 | 23 |
|  |  | Brand 2 | 29 |
| Fried pulses | This is a type of pre-packaged, fried snack made from peas or mung beans. The packaging typically includes the brand name, producer's name, production and expiration dates, and a list of ingredients. Some packages also include nutrition information. | Brand-1 (pulse) | 31 |
| Fried peas | This is a type of pre-packaged, fried snack made from peas or mung beans. The packaging typically includes the brand name, producer's name, production and expiration dates, and a list of ingredients. Some packages also include nutrition information. | Brand-1 (pea) | 21 |
| Biscuit | Biscuits are popular forms, these are mass-produced by a large factory and sold across the country under a specific brand name. The packaging contains information such as the name of the manufacturer, production date, and expiry date. Additionally, the label lists the ingredients used in the product along with some nutritional information. | Brand-1 | 30 |
|  |  | Brand-2 | 30 |
|  |  | Brand-3 | 30 |
|  |  | Brand-4 | 30 |
|  |  | Brand-5 | 23 |
|  |  | Brand-6 | 26 |
| Milk Chocolate | It is a solid chocolate confectionery containing cocoa, sugar and milk. | Brand 1 | 18 |
| lozenge | Lozenges are one of the oldest forms of [sugar confectionery](https://www.sciencedirect.com/topics/food-science/sugar-confectionery). Lozenges are cut from a sheet of dough and then dried. Lozenges are made by taking a milled sugar and making it into a dough. | Brand 1 | 15 |
|  |  | Brand_2 | 27 |
| Lillipop | Lozenges are also another kind of sugar confectionaries | Brand_1 | 22 |
| Ice-cream | It is a soft, sweet frozen food made with milk and cream and typically flavoured with vanilla, fruit, or other ingredients. | Brand_1 | 25 |
|  |  | Brand_2 | 17 |
|  |  | Brand_3 | 12 |

### Adapted from Choudhury et.al.,2021

**Supplementary Table 2: Contribution of per serving of food to the daily intake of SFA, TFA, sodium, sugar**

|  | Serving size on label | Serving size (g) | Amount of the constituents (g)/serving^1^  (Percent contribution to a healthy diet/serving)^2^ | | | |
| --- | --- | --- | --- | --- | --- | --- |
|  |  |  | SFA | TFA | sodium | sugar |
| Chips | Brand 1 | 15 | 1.88(8.44) | 0.02(0.68) | 0.14(6.26) | 1.08(4.32) |
|  | Brand 2 | 22 | 2.82(12.67) | 0.02(1.1) | 0.08(3.44) | 1.61(6.42) |
|  | Brand 3 | 25 | 2.35(10.58) | 0.03(1.36) | 0.29(12.61) | 1.76(7.04) |
| Chanachur | Brand 1 | 28 | 4.17(18.77) | 0.03(1.4) | 0.04(1.95) | 2.07(8.29) |
|  | Brand 2 | 28 | 4.07(18.33) | 0.03(1.53) | 0.26(11.2) | 4.87(19.49) |
|  | Brand 3 | 28 | 5.06(22.77) | 0.03(1.53) | 0.27(11.69) | 2.94(11.76) |
| Noodles | Brand 1 | 15.5 | 1.91(8.58) | 0.02(1.13) | 0.09(4.04) | 0.96(3.84) |
|  | Brand 2 | 15.5 | 0.01(0.03) | 0(0.01) | 0.17(7.55) | 0.91(3.66) |
| Fried Peas | Brand 1 | 20 | 1.18(5.31) | 0.01(0.45) | 0.4(17.43) | 0.81(3.22) |
| Fried pulse | Brand 1 | 25 | 1.98(8.89) | 0.02(1.02) | 0.72(31.3) | 0.6(2.4) |
| Biscuits | Brand 1 | 50 | 4.65(20.93) | 0.03(1.36) | 0.64(27.83) | 13.52(54.08) |
|  | Brand 2 | 18 | 2.45(11.02) | 0.02(0.98) | 0.14(5.92) | 1.76(7.06) |
|  | Brand 3 | 18 | 2.25(10.13) | 0.07(3.19) | 0.04(1.6) | 5.04(20.16) |
|  | Brand 4 | 60 | 4.68(21.06) | 0.07(3) | 0.22(9.5) | 10.5(42) |
|  | Brand 5 | 70 | 4.68(21.06) | 0.03(1.27) | 0.59(25.57) | 11.34(45.36) |
|  | Brand 6 | 40 | 7.77(34.96) | 0.03(1.45) | 0.11(4.94) | 7.16(28.64) |
| Milk chocolate | Brand 1 | 13.2 | 0.21(0.95) | 0.03(1.36) | 0.12(5.05) | 1.28(5.11) |
| Lozenge | Brand 1 | 3 | 0(0) | 0(0) | 0(0.08) | 1.49(5.98) |
|  | Brand 2 | 3 | 0(0) | 0(0) | 0.01(0.42) | 2.13(8.52) |
| Lollipop | Brand 1 | 6 | 3.45(15.5) | 0(0.16) | 0(0.15) | 0.98(3.91) |
| Chutney | Brand 1 | 20 | 0.3(1.35) | 0.01(0.36) | 0.22(9.39) | 1.86(7.44) |

(SFA=Saturated fatty acid, TFA= Trans fatty acid)
 ^1^The serving size was estimated from the declaration provided on the packet. However, where the declaration was absent, the lowest value of the same group of product was utilized;

^2^Percent contribution of one serving of analyzed foods to allowable the intake of SFA (200kcal; 10% of the energy), TFA (2.2 g; 1% of the energy) sodium (2.3g or 2300 mg), and sugar (25g/day, 5% of the energy) of a 2000 kcal/day diet recommend by World Health Organization (WHO) (WHO, 2020).
